# Kin discrimination in plants: overview and implications for population and community ecology

**DOI:** 10.1002/brv.70194

**Published:** 2026-06-18

**Authors:** Akira Yamawo

**Affiliations:** ^1^ Center for Ecological Research, Kyoto University 2‐509‐3, Hirano Otsu Shiga 520‐2113 Japan

**Keywords:** competition, cooperation, coexistence, plant behaviour, plant community, population genetics, spatial structure, plant‐herbivore interactions, genetic diversity, species diversity

## Abstract

Following the discovery of identity discrimination, particularly self and kin discrimination amongst plant competitors, research on interplant interactions has advanced significantly within plant physiology and evolutionary ecology. This review synthesizes current knowledge on how both self and kin discrimination influence plant growth, resource allocation, defence, and reproduction. I highlight their ecological and evolutionary consequences for population dynamics, community structure, and multi‐trophic interactions. A key insight is that, under some circumstances, identity discrimination may shape the maintenance or erosion of genetic diversity by modulating competitive interactions and spatial genetic structure within populations. Finally, I present several open questions, such as how identity discrimination operates across gradients of plant competition and trophic levels, and propose a few testable hypotheses and experimental approaches regarding the role of competitive identity discrimination under natural settings.

## I. INTRODUCTION

Plants rarely grow in isolation but instead coexist with neighbours, where interactions such as competition and facilitation strongly influence their performance and evolution (Callaway & Walker, 1997; Brooker *et al*., 2008). Plants may encounter not only heterospecifics, but also unrelated conspecifics, or genetically related individuals such as kin or clonal ramets. Clonal reproduction is particularly common, with clonal plants dominating many ecosystems (Fischer & van Kleunen, 2001; Herben & Klimešová, 2020); for instance, about 70% of temperate forest understory species are clonal (van Groenendael & de Kroon, 1990). Natural populations often exhibit spatial genetic structure (Argyres & Schmitt, 1992; Vekemans & Hardy, 2004), creating consistent interactions between related individuals. Theoretical models suggest that such population viscosity can promote the evolution of traits whose expression depends on neighbour relatedness, because interactions are more likely to occur among relatives (Kanwal & Gardner, 2022). Under these conditions, kin selection theory predicts the evolution of phenotypic plasticity in response to the relatedness of neighbours – a mechanism defined as kin recognition (Hamilton, 1964; Hepper, 1986; Waldman, 1988; Green *et al*., 2024). Consequently, plants have been observed to adjust traits such as growth, root allocation, and leaf positioning in response to conspecific neighbours (Mahall & Callaway, 1991; Chen, During & Anten, 2012; Novoplansky, 2019; Yamawo & Mukai, 2020; Sher *et al*., 2025). This plasticity in potentially adaptive traits is frequently interpreted as evidence of kin recognition, suggesting that plant–plant interactions are not fixed but are instead modulated by the relatedness of interacting individuals. Thus, plant evolution can be shaped by interactions among relatives, and the effects of an individual's traits cannot be understood in isolation but only in the context of neighbour relatedness (e.g. Riechert & Hammerstein, 1983; Cabal *et al*., 2020).

Plant–plant interactions are well known as key drivers of the material cycles and productivity in ecosystems, as are interactions with herbivory and predatory insects (Agrawal *et al*., 2006; Novoplansky, 2009; Chen *et al*., 2012; Yamawo, 2021; Ohsaki *et al*., 2022a; Gols & Harvey, 2023). Therefore, differential self/non‐self (S/NS) and kin/non‐kin interactions in plants are hypothesized to play important roles not only in the performance and fate of individual plants, but also in ecological processes across broader biological scales, including population, community, ecosystem, and evolutionary levels (Herben & Novoplansky, 2008; Dudley, Murphy & File, 2013; Yamawo & Mukai, 2020; Anten, & Chen, 2021).

Over the past 20 years, attention has focused strongly on the question of whether plants can differentiate self or kin from non‐self and non‐kin (Dudley *et al*., 2013; Novoplansky, 2019). Early investigations into responses to S/NS neighbours in plants were conducted in *Fragaria chiloensis* (Holzapfel & Alpert, 2003) and *Pisum sativum* (Falik *et al*., 2003). In those studies, the roots of neighbouring plants were grown in shared pots, alongside either roots from another part of the same plant (self) or roots of a different plant of the same species (non‐self). Their findings suggest that while total biomass production was similar in self and non‐self neighbourhoods, plants allocated a larger proportion of their biomass to root growth under non‐self conditions. However, in some experiments, the neighbour identity was confounded with other factors such as per‐root soil volume and nutrient availability (Schenk, 2006; Hess & De Kroon, 2007). Furthermore, differential responses to self and non‐self neighbours were not detected in certain plant species, including *Avena sativa* (Semchenko, Hutchings & John, 2007*a*), *Andropogon gerardii* (Markham & Halwas, 2011), *Glechoma hederacea*, and *Fragaria vesca* (Semchenko, John & Hutchings, 2007*b*). In spite of these methodological challenges, phenotypic responses to neighbour identity, potentially reflecting an adaptive strategy to distinguish self from non‐self, have been reported in both belowground and aboveground experiments. However, because pot volume (*V*), nutrient amount (*N*), and nutrient concentration (*C*) are mathematically linked (*C* = *N*/*V*), no pot‐based design can fully control for all three simultaneously. Thus, while pot experiments necessarily involve trade‐offs, the consistency of S/NS‐dependent responses across studies suggests that such discrimination may influence plant behaviour and interplant interactions, albeit in a context‐dependent manner.

In addition to studies of S/NS discrimination, researchers have investigated whether plants exhibit phenotypic plasticity in response to the relatedness of neighbouring individuals. For example, Dudley & File (2007) showed that plants demonstrate phenotypic plasticity in morphology, architecture, and other potentially adaptive phenotypic traits in response to neighbour genetic relatedness. Most of these studies show that plants can plastically modify their morphology and architecture depending on neighbour identity and relatedness (Table 1), suggesting that plants exhibit relatedness‐dependent phenotypic plasticity that may result in reduced competitive intensity among self or kin. This response pattern is broadly referred to as kin discrimination. Crucially, the observation of kin‐recognition does not imply that kin selection has been demonstrated, because plastic responses to neighbour relatedness may arise for multiple evolutionary reasons (Hamilton, 1964; Hepper, 1986; Waldman 1988). While phenotypic plasticity in response to neighbors is widely documented, demonstrating that these responses are driven by kin selection requires a more comprehensive understanding of their fitness consequences (Hamilton, 1964; Hepper, 1986; Waldman, 1988; Green *et al*., 2024). Currently, many studies lack data on the direct and indirect fitness effects of observed traits (but see Kelly, 1996; Yamawo, Sato & Mukai, 2017; Yamawo & Mukai, 2020; Xu *et al*., 2024). In addition, despite a few hypotheses (Novoplansky, 2009; Wuest *et al*., 2022), the mechanisms underlying competitive identity discrimination in plants are yet to be deciphered. To fully align these observations with kin selection theory, it is essential to investigate inclusive fitness outcomes and the underlying physiological or genetic mechanisms of discrimination (Hamilton, 1964; Hepper, 1986; Waldman, 1988). Some compounds and genetic regions responsible for kinship discrimination in plants have recently been identified and reviewed by Anten & Chen (2021) and Mazal *et al*. (2023).

**Table 1:** Summary of kin and self‐discrimination studies. These studies were identified through literature searches using Web of Science and Google Scholar with keywords such as ‘kin recognition’, ‘kin discrimination’, and ‘self/non‐self recognition’ in plants. Peer‐reviewed experimental studies reporting behavioural or growth responses to kin versus non‐kin were included, covering publications from 1990 to 2025, together with selected unpublished data from the author. Note that in this table, ‘self‐discrimination’ and ‘kin discrimination’ are categorized separately to distinguish between responses to genetically identical ramets (self) and distinct individuals within the same population (kin), although these interactions are treated as a continuum of plasticity to relatedness throughout the main text. For some plant species, I additionally include unpublished data from the authors.

Plant species	Life style and growth form	Discrimination	References
**Kin discrimination**			
**Apiaceae**			
*Peucedanum palustre*	Perennial herb	Absence	Lepik *et al*. (2012)
**Amaranthaceae**			
*Alternanthera philoxeroides*	Perennial herb	Presence	Zhang *et al*. (2019)
*Chenopodium quinoa*	Annual herb	Presence	Sher *et al*. (2023, 2024)
**Asteraceae**			
*Achillea millefolium*	Perennial herb	Absence	Lepik *et al*. (2012)
*Ambrosia dumosa*	Perennial shrub	Presence	Mahall & Callaway (1996)
*Ambrosia artemisiifolia*	Annual herb	Presence	File *et al*. (2012)
*Artemisia tridentata*	Perennial shrub	Presence	Karban *et al*. (2013)
*Helianthus tuberosus*	Perennial herb	Presence	Fukano *et al*. (2019)
*Leontodon hispidus*	Perennial herb	Absence	Lepik *et al*. (2012)
*Taraxacum officinale*	Perennial herb	Presence	Lee *et al*. (2021)
*Taraxacum platycarpum*	Perennial herb	Presence	Lee *et al*. (2021)
*Xanthium italicum*	Annual herb	Presence	Abd El‐Gawad *et al*. (2017)
**Balsaminaceae**			
*Impatiens capensis*	Annual herb	Absence	Argyres & Schmitt (1992)
*Impatiens pallida*	Annual herb	Presence	Murphy & Dudley (2009)
**Brassicaceae**			
*Cakile edentula*	Annual herb	Presence	Dudley & File (2007); Donohue (2003)
*Arabidopsis thaliana*	Annual herb	Presence	Biedrzycki *et al*. (2010); Crépy & Casal (2015)
*Moricandia moricandioides*	Annual herb	Presence	Torices *et al*. (2018)
*Raphanus sativus var. sativus*	Annual herb	Presence	Yamawo, A. (unpublished)
**Caryophyllaceae**			
*Lychnis flos‐cuculi*	Perennial herb	Presence	Lepik *et al*. (2012)
**Convolvulaceae**			
*Ipomoea hederacea*	Annual vine	Presence	Biernaskie (2011)
**Crassulaceae**			
*Kalanchoë daigremontiana*	Perennial succulent herb	Presence	Yamawo *et al*. (2017)
**Cycadaceae**			
*Cycas edentata*	Perennial cycad tree	Presence	Marler (2013)
**Ebenaceae**			
*Diospyros morrisiana*	Evergreen tree	Presence	He *et al*. (2021)
**Euphorbiaceae**			
*Mallotus japonicus*	Deciduous tree	Presence	Yamawo (2015)
**Fabaceae**			
*Trifolium repens*	Perennial herb	Presence	Lepik *et al*. (2012)
*Medicago lupulina*	Perennial herb	Absence	Lepik *et al*. (2012)
*Medicago minima*	Annual herb	Presence	Ehlers *et al*. (2016); Tomiolo *et al*. (2021)
*Lupinus angustifolius*	Annual herb	Absence	Milla *et al*. (2012)
*Glycine max*	Annual herb		Murphy *et al*. (2017)
**Fagaceae**			
*Fagus crenata*	Deciduous tree	Presence	Takigahira & Yamawo (2019)
**Malvaceae**			
*Abutilon theophrasti*	Annual herb	Presence	Willson *et al*. (1987)
*Urena lobata*	Perennial shrub	Presence	Yamawo (2021)
**Myrtaceae**			
Eucalyptus urophylla	Evergreen tree	Presence	Bertoli *et al*. (2020)
**Phrymaceae**			
*Erythranthe guttata*	Perennial herb	Presence	Lewis & Friedman (2025)
**Pinaceae**			
*Pseudotsuga menziesii*	Evergreen tree	Presence	Pickles *et al*. (2017)
**Poaceae**			
*Sorghum vulgare*	Annual grass	Presence	Zhang *et al*. (2016)
*Deschampsia cespitosa*	Perennial grass	Presence	Lepik *et al*. (2012); Semchenko *et al*. (2014)
*Echinochloa crus‐galli*	Annual grass	Presence	Ding *et al*. (2023)
*Oryza sativa*	Annual grass	Presence	Yang *et al*. (2018)
*Pseudoroegneria spicata*	Perennial grass	Presence	Yang *et al*. (2015)
*Phalaris arundinacea*	Perennial grass	Presence	Collins *et al*. (2018)
*Phleum pratense*	Perennial grass	Absence	Lepik *et al*. (2012)
*Triticum aestivum*	Annual grass	Presence/absence	Saleh & Kniss (2022); Mahal *et al*. (2023)
*Zea mays*	Annual grass	Presence	Zhou *et al*. (2022)
*Triplasis purpurea*	Annual grass	Presence	Cheplick & Kane (2004)
*Hordeum vulgare*	Annual	Presence	Ninkovic (2003)
**Phytolaccaceae**			
*Phytolacca americana*	Perennial herb	Presence	Willson *et al*. (1987)
**Plantaginaceae**			
*Plantago lanceolata*	Perennial herb	Absence	Tonsor (1989)
*Plantago asiatica*	Perennial herb	Presence	Yamawo & Mukai (2017, 2020)
**Polygonaceae**			
*Rumex obtusifolius*	Perennial herb	Presence	Sakurai, T. & Yamawo, A. (unpublished)
**Lamiaceae**			
*Glechoma hederacea*	Perennial creeping herb	Presence	Goddard *et al*. (2020)
*Glechoma longituba*	Perennial creeping herb	Presence	Fan *et al*. (2022)
**Rosaceae**			
*Potentilla anserina*	Perennial creeping herb	Absence	Rautiainen *et al*. (2004)
**Solanaceae**			
*Solanum mauritianianum*	Perennial shrub	Presence	Willson *et al*. (1987)
*Solanum lycopersicum*	Perennial herb	Absence	Willson *et al*. (1987)
**Violaceae**			
*Viola tricolor*	Annual herb	Presence	Lankinen (2008)
**Zygophyllaceae**			
*Larrea tridentata*	Perennial shrub	Presence	Mahall & Callaway (1991)
**Self discrimination**			
**Asteraceae**			
*Ambrosia dumosa*	Perennial shrub	Presence	Mahall & Callaway (1991, 1996)
*Artemisia tridentata*	Perennial shrub	Presence	Karban & Shiojiri (2009)
**Aizoaceae**			
*Carpobrotus edulis*	Perennial creeping herb	Presence	Roiloa *et al*. (2014)
**Fabaceae**			
*Trifolium repens*	Perennial herb	Presence	Falik *et al*. (2006)
*Pisum sativum* var. *arvense*	Annual climbing herb	Presence	Falik *et al*. (2003)
**Poaceae**			
*Buchloe dactyloides*	Perennial grass	Presence	Gruntman & Novoplansky (2004)
**Vitaceae**			
*Cayratia japonica*	Perennial vine	Presence	Fukano & Yamawo (2015)
**Cucurbitaceae**			
*Monordica charantia var. pavel*	Annual vine	Presence	Sato *et al*. (2018)
*Cucumis sativus*	Annual vine	Absence	Sato *et al*. (2018)
**Passifloraceae**			
*Passiflora caerulea*	Perennial vine	Presence	Sato *et al*. (2018)
**Rosaceae**			
*Fragaria chiloensis*	Perennial creeping herb	Presence	Holzapfel & Alpert (2003)
*Potentilla reptans*	Perennial creeping herb	Presence	Chen *et al*. (2015)

Despite the fact that the complete mechanisms underlying such individual identity discrimination remain poorly understood, diverse forms of phenotypic plasticity in response to genetic relatedness among plants clearly exist and have the potential to influence community‐ and ecosystem‐level processes. Indeed, accumulating evidence indicates that plastic responses of plants to competitors can scale up from individual interactions to influence community‐level processes (Hess *et al*., 2022; Gibbs, Levine & Turcotte, 2025). In this review, I use the term ‘kin discrimination’ to refer to the expression of plastic phenotypic responses to neighbour relatedness, including S/NS, regardless of whether the underlying recognition mechanisms or fitness consequences have been fully elucidated. In the following, I discuss the effects of kin discrimination on plant interactions at the population and community levels and suggest potential future research directions. The terminology used to describe these identity‐based interactions is summarized in Table 2.

**Table 2:** Definitions of key terms used in this review.

Term	Definition as used in this review
Genet	The total number of genetic individuals originating from a single fertilization event (i.e. seed‐derived individual), which may consist of one or multiple ramets produced through clonal growth.
Ramet	A potentially physiologically independent modular unit produced through clonal growth; a component of a genet that may function as an individual in ecological interactions.
Clonal growth	A form of vegetative reproduction in which genetically identical ramets are produced and spatially distributed from a single genet.
Kin discrimination (Kin recognition)	The ability of a plant to express phenotypic plasticity in response to the genetic relatedness of neighbours, *encompassing the differentiation between self (genetically identical ramets of the same genet) and non‐self individuals*. In this review, following current evolutionary theory, these terms are used synonymously to describe both the recognition mechanism and the observable outcome.
Identity discrimination	A broad concept referring to the ability of plants to distinguish among interaction partners based on genetic relatedness (*ranging from self and kin to non‐kin*) or species identity.
Population structure	The spatial, genetic, and demographic organization of individuals within a population, including patterns of relatedness, clonal structure, and spatial arrangement.
Community‐level effects	The consequences of identity discrimination at the individual or population level for interspecific interactions, species coexistence, and overall community structure.

## II. THE PREVALENCE OF COMPETITIVE IDENTITY DISCRIMINATION

### (1) Taxonomic distribution and phylogenetic patterns

The prevalence of kin discrimination has been reported or observed in 66 plant species in 58 genera of 30 families (Table 1). Reports indicate its occurrence across various life histories and growth forms, including annuals, perennials, and trees (both pioneers and climax species). This broad but patchy distribution aligns with recent global syntheses suggesting that kin discrimination is a phylogenetically widespread trait that has evolved independently across diverse lineages in the tree of life (Green *et al*., 2024). Among the 66 species that have been studied, 54 species in 29 families have shown some form of S/NS or kin discrimination, with the type and intensity of the response changing in relation to how genetically similar the neighbouring plants are (Table 1). Despite initial reluctance among scholars, kin discrimination has gradually gained acceptance (Pennisi, 2019). This reluctance stemmed from several factors: the long‐standing assumption that plants, unlike animals, lack the capacity for recognition; the difficulty of experimentally disentangling kin effects from resource competition or environmental variation; doubts about whether kin discrimination could provide sufficient inclusive fitness benefits to be evolutionarily stable; and a general resistance to applying concepts of social behaviour, traditionally developed in animal studies, to plants. However, theories of social behaviour do not inherently require highly complex cognition or centralized nervous systems, and have been shown to apply broadly to organisms with decentralized or alternative forms of information processing, including fungi and other non‐neural systems (Hamilton, 1964; West, Griffin & Gardner, 2007; Bastiaans, Debets & Aanen, 2016). With the accumulation of empirical evidence across diverse taxa and the integration of kin selection theory into plant ecology, however, kin discrimination has gradually gained wider acceptance. In some species, such as *Arabidopsis thaliana*, leaf overlap with kin is reduced, thus reducing light competition with relatives (Crépy & Casal, 2015). Sea rocket (*Cakile edentula* var. *lacustris*) plants increase root allocation, presumably enhancing below‐ground greater competitive effort if neighbours are non‐kin plants (Dudley & File, 2007). Although evidence of kin discrimination in plants is increasing, most of the published studies have been conducted with herbaceous species, with some studies reporting it in tree species such as *Cycas edentata*, *Mallotus japonicus*, *Pseudotsuga menziesii*, and *Eucalyptus urophylla* (Table 1; Marler, 2013; Yamawo, 2015; Pickles *et al*., 2017; Takigahira & Yamawo, 2019; Bertoli *et al*., 2020).

### (2) Environmental contingencies of identity discrimination

Competitive kin discrimination depends on the environmental conditions. For example, root activity and nutrient uptake were lower in sibling pairs of *Sorghum* than in non‐sibling pairs but only under low nutrient availability (Li, Xu & Feng, 2018). Palmer *et al*. (2016) showed that limitations of N and P are key to determining this effect. They grew *Arabidopsis thaliana* seedlings alone, paired with a sibling, a relative from a different mother in the same accession, or a stranger from a different accession and found that plants grew more lateral roots when grown with alien plants, but only when the nutrient concentration was below 75% of that required for plant growth (Palmer *et al*., 2016). Similarly, relative‐ and sibling‐paired plants developed more lateral roots than single plants under low nutrient availability. Moreover, the effects of kin discrimination on the performance of plants can be density dependent. Theoretical work has demonstrated that density‐dependent factors, such as dispersal constraints and population viscosity, are critical in determining whether altruistic or competitive behaviors among kin are favoured by selection (Kanwal & Gardner, 2022). For example, Lepik *et al*. (2012) showed that the inflorescence biomass of *Trifolium repens* increases with increasing neighbour density only in sibling groups, although petiole length (a key trait related to competition for light) was positively correlated with increasing neighbour density only in non‐sibling groups, reflecting how kin‐discrimination can affect the resource allocation trade‐off between competitive and reproductive functions.

The main environment for plants is typically other plants, and kin‐discrimination can be strongly influenced by the identity and composition of neighbouring individuals. However, only a few previous studies have tested kin discrimination under more natural conditions, examining plant responses to kin in the presence of both conspecific strangers and heterospecifics (Waldman, 1988). For example, *Plantago asiatica* seeds germinate faster and more synchronously in the presence of *T. repens* seeds, but only when siblings are present, whereas the presence of non‐sibling *P. asiatica* seeds does not trigger the same response (Yamawo & Mukai, 2017). These results show that *P. asiatica* seeds integrate information about the relatedness of neighbouring conspecifics and the presence of heterospecific seeds through water‐soluble chemicals. Such rapid and synchronized germination may promote interactions among siblings and enhance suppression of heterospecific seedlings during early growth stages (Yamawo & Mukai, 2020). Similarly, the effects of kin discrimination in other species can be context‐dependent: *Medicago rigidula* exhibited higher survival under intra‐genotype than inter‐genotype competition, but only when grown in soils collected beneath allelopathic thyme (*Thymus vulgaris*) shrubs, whereas *Medicago minima* showed reduced kin‐competition only when grown in non‐thyme soils (Ehlers *et al*., 2016). These studies highlight that kin‐discrimination is modulated by the composition and identity of neighbouring plants, illustrating the importance of environmental contingencies in shaping plant social responses.

Although studies of kin discrimination have been conducted in various plant species, many of its environmental contingencies are still poorly understood. These studies are biased towards herbs, mostly in the seedling stage. As zoochory often implies that seeds are dispersed to the same locations with their genetically related siblings (e.g. Beckman & Rogers, 2013; Isla *et al*., 2024), kin discrimination is likely to be more widespread in zoochorous and other plants in which sibling seeds are dispersed together especially if they do not exhibit germination asynchrony (Terakawa *et al*., 2009), but as of today, available data remain largely missing.

The variable distribution of kin discrimination across congeners and among species within the same family suggests that this trait is not phylogenetically conserved, but instead may arise under specific ecological contexts, potentially reflecting the action of local selective pressures. To properly evaluate the adaptive effects of kin‐discrimination, data must be collected and analysed across sufficiently diverse phylogenetic groups, large and diverse geographic regions, and under diverse abiotic and biotic conditions.

## III. EFFECTS ON POPULATION GROWTH AND GENETIC STRUCTURE

S/NS and kin discrimination can influence population‐level properties by shaping, though not exhaustively, the following processes: (*i*) the intensity of intraspecific competition, (*ii*) stress tolerance mediated by interplant communication, and (*iii*) the development of reproductive traits.

### (1) Intraspecific competition

Some studies have found that in clonal plants that exhibit decreased competition with clone mates, self‐pairs produced more seeds and had greater clonal reproduction and allocation than non‐self pairs (Yamawo *et al*., 2017; Yang *et al*., 2018). For example, *Kalanchoë daigremontiana* competing with clone mates increased allocation to clonal reproduction compared to plants competing with unrelated neighbours (Yamawo *et al*., 2017). This difference in reproductive allocation appears to be driven by root competition, with plants competing against non‐self neighbours allocating more to roots and less to reproduction. Additionally, root exudates from these clonal plants reduced both root growth and clonal reproduction in non‐self neighbours, i.e. expressed increased allelopathic response towards non‐kin neighbours (Yamawo *et al*., 2017). These results suggest that, in the short term, populations composed solely of clones may exhibit higher population growth than non‐clonal populations. A similar trend had been predicted by an earlier simulation model demonstrating that S/NS discrimination in clonal plants could significantly increase spatial aggregation and decrease clonal diversity (Herben & Novoplansky, 2008). Although empirical data on the population‐level consequences of S/NS and kin discrimination remain limited, theoretical and conceptual studies have hypothesized that strong S/NS discrimination could influence fine‐scale genetic structure within populations (Herben & Novoplansky, 2008). These hypotheses highlight the need for longer‐term, population‐level empirical studies to evaluate whether and under what conditions such effects on genetic diversity occur. Like S/NS discrimination, kin discrimination also contributes to increased seed production through the avoidance of competition between kin individuals (Crépy & Casal, 2015; Yang *et al*., 2018). However, these studies focused on the adaptive significance, and thus the effects on subsequent population growth rates remain unclear. Future experiments conducted over generational time scales may allow us to evaluate how phenomena at the population level are influenced by the dependence of competition reduction on kin discrimination.

### (2) Interplant stress communication

Many plants respond to both biotic and abiotic stresses by perceiving and transmitting stress‐related cues. In response to abiotic stress, some plants release volatile organic compounds, which can prime neighbouring plants for stress tolerance (Loreto & Schnitzler, 2010; Possell & Loreto, 2013). Stress‐related communication has also been observed in root systems. For instance, unstressed *Pisum sativum* plants rapidly close their stomata upon detecting cues emitted from the roots of drought‐stressed or abscisic acid–treated neighbours. These responses can propagate to additional unstressed plants further from the initial stress source, highlighting the potential for stress cues to affect plant populations at larger spatial scales (Falik *et al*., 2011, 2012; Falik, Hoffmann & Novoplansky, 2024; Falik & Novoplansky, 2023).

The effectiveness of interplant communication may depend on the genetic relatedness and population structure of the interacting organisms (Karban & Shiojiri, 2009; Karban *et al*., 2013; Ito *et al*., 2025). Previous studies suggest that intra‐ and interplant signalling can reduce competition, not only between different organs within the same plant (Mahall & Callaway, 1991; Falik *et al*., 2003; Falik, de Kroon & Novoplansky, 2006; Gruntman & Novoplansky, 2004; Fukano & Yamawo, 2015) but also among clone mates and genetically related individuals within species, a pattern that has been documented across multiple plant taxa (Dudley & File, 2007; Yamawo, 2015, 2021; Yamawo *et al*., 2017). In *Artemisia tridentata*, for example, plants exposed to volatiles collected from relatives, relative to plants exposed to volatiles collected from strangers, showed more pronounced volatile‐mediated programming (Karban & Shiojiri, 2009; Karban *et al*., 2013). Similarly, 60 min after the onset of salt stress, neighbouring *Plantago asiatica* plants sharing the same soil closed more stomata when the stressed plant was genetically closer (e.g. a sibling or from a nearby population) than plants from more distant populations (Ito *et al*., 2025). In contrast, aboveground stress signalling was equally effective regardless of genetic relatedness (Ito *et al*., 2025). These findings suggest that kin discrimination might play a crucial role in interplant stress communication.

If stress‐cue transmission and effectiveness depend on kin discrimination (Waldman, 1988), such processes may influence population dynamics by affecting survival under stressful conditions according to relatedness. However, empirical evidence linking kin discrimination to population‐level genetic diversity remains limited. Rather than universally decreasing genetic diversity, discrimination processes primarily contribute to the spatial and demographic structuring of populations, particularly at fine spatial scales (Herben & Novoplansky, 2008). Under these structured conditions, kin discrimination can help maintain or even promote the persistence of genetic diversity in traits associated with altruistic behaviours, whereas other aspects of genetic diversity may still decline depending on dispersal, ecological context, and selective pressures (Scott, Grafen & West, 2022; Kanwal & Gardner, 2022). Thus, the effects of kin discrimination on genetic diversity are context‐dependent, reflecting the interaction between kin structure, dispersal mechanisms, and selective dynamics. This context‐dependency remains a central theme in recent broad‐scale syntheses of kin discrimination across the tree of life, emphasizing that the functional significance of recognition can vary dramatically depending on the life history and ecological constraints of the species (Hepper, 1986; Green *et al*., 2024). These effects on the population can be tested through field surveys and experiments, where kin structure and genetic relatedness among neighbouring plants will be assessed and manipulated. Specifically, genetic diversity in plant populations growing under saline stress may be reduced for neutral or non‐altruistic traits, but maintained or enhanced for traits whose expression benefits neighbouring relatives in kin‐structured populations. In addition, experimental manipulation of kin structure under salt stress conditions would also be effective in testing these predictions.

### (3) Reproductive traits

Several studies have demonstrated kin discrimination in flower traits (Torices, Gómez & Pannell, 2018; Torices *et al*., 2021). *Moricandia moricandioides* modifies larger floral displays or floral colour when growing with kin than non‐kin neighbours (Torices *et al*., 2018; Torices *et al*., 2021). A prominent floral display can enhance pollinator attraction at the patch level, benefiting both the plant and its neighbours, a phenomenon known as the ‘magnet effect’ (Laverty, 1992; Essenberg, 2012). However, an increase in floral display size may reduce neighbouring plants' pollination success, as pollinators tend to concentrate on the most conspicuous flowers (Mitchell *et al*., 2004; Grindeland, Sletvold & Ims, 2005; Ishii & Harder, 2006; Glaettli & Barrett, 2008). Therefore, when plants grow among kin, the evolutionary advantage of producing a larger floral display depends on the spatial resolution of pollinator preferences for more visually attractive flowers, on the local and global abundances of flowers with variable display quality and size, and the level of competition for pollinators.

Kin discrimination can increase intraspecific phenotypic variation in floral display size and seed production through plastic responses to neighbour relatedness. By contrast, kin selection acting on such variation may, under certain conditions, reduce the genetic diversity of plant populations. Tomizuka, Tachiki & Yamawo (2025) developed evolutionary simulations to examine two floral strategies that plants adopt depending on the genetic relatedness of neighbouring plants. They categorized the evolutionary outcomes of floral display size into four types, based on whether the display size increased or decreased relative to plants lacking kin discrimination. A key result was that plants growing among kin were expected to produce larger floral displays, whereas those coexisting with unrelated individuals were selected to selfishly reduce their floral displays, patterns consistent with field observations made by Torices *et al*. (2018). However, these predictions are based on simplified assumptions about pollinator behaviour, particularly that pollinators primarily choose among spatially separated groups of plants and then visit flowers largely at random within a group. In natural systems, pollinators often show fine‐scale selectivity among individual flowers, influenced by floral traits, reward distribution, and learning. Moreover, floral traits that exhibit kin discrimination, and the resulting evolution of floral display size had spill‐over effects at the population level. Such kin‐dependent expression of floral display size and seed production generally increased the intraspecific variation in the floral display size and seed production and decreased the genetic diversity of populations (Tomizuka *et al*., 2025). However, although this model cited in this section suggests that kin discrimination can alter the genetic diversity within a population through changes in interaction with pollinators, the specific effects remain to be explored in studies using actual pollinators.

## IV. EFFECTS OF KIN‐DISCRIMINATION ON THE BIOLOGICAL COMMUNITY

### (1) Plant community

Recent evidence suggests that kin‐discrimination can affect interspecific interactions. In competition with *T. repens*, sibling pairs of *P. asiatica* orient more leaves toward *T. repens* than to conspecific neighbours, increasing the competitive effects of *P. asiatica* against *T. repens* (Yamawo & Mukai, 2020). In addition, *P. asiatica* discriminates kin during germination (Yamawo & Mukai, 2017): in the presence of *T. repens* seeds, *P. asiatica* germinates faster when neighbouring seeds are sibling than non‐sibling. The acceleration of germination may provide a competitive advantage in the seedling stage (Orrock & Christopher, 2010; Ohsaki, Mukai & Yamawo, 2020). These results indicate that, at least under conditions where the heterospecific competitor *T. repens* is present, interactions among kin individuals are facilitative (Yamawo & Mukai, 2020).

Xu *et al*. (2021) examined possible mechanisms through which kin individuals may facilitate each other during interspecific competition in rice (*Oryza sativa*). They focused on three mechanisms: changes in root behaviour, allelopathic effects (growth suppression by chemicals), and resource partitioning. They found that mixtures of kin plants inhibit the establishment and growth of paddy weeds more vigorously than mixtures of non‐kin plants. Importantly, increased genetic relatedness enhanced production and allocation of root growth towards neighbouring weeds (Xu *et al*., 2021). These results are consistent with studies of *Plantago* spp. (Yamawo & Mukai, 2020); relatedness allows plants to discriminate between neighbouring collaborators (self, kin) and competitors and to adjust their growth, competitiveness, and chemical production.

Furthermore, the ecological consequences of kin discrimination are not limited to herbaceous plants but also extend to long‐lived woody species in complex forest ecosystems. In interior Douglas‐fir, *Pseudotsuga menziesii*, seedling size and morphological traits are significantly influenced by both neighbourhood relatedness and species composition (Asay, Simard & Dudley, 2020). These responses were context‐dependent, with the effects of kin presence varying depending on whether seedlings grew in conspecific or mixed‐species neighbourhoods. This suggests that kin discrimination is an important factor interacting with broader community composition to shape the productivity and structural development of forest stands. Consistent with this perspective, previous work has also shown that seedling survival rates can vary depending on their genetic relatedness to neighbouring conspecific adult trees, indicating that kin structure may influence demographic processes in forest populations (Akaji *et al*., 2016).

In contrast to the competitive advantage under interspecific competition, *P. asiatica* siblings have been demonstrated to be vulnerable to intraspecific competition from other genotypes (Yamawo, A., unpublished data). Although the detailed mechanisms remain unclear, this pattern may result from cooperative behaviour among sibling individuals but more selfish behaviour among non‐siblings (Yamamichi *et al*., 2020). Regardless of the mechanism, this possibility suggests a novel scheme of sequential consequences in plant community dynamics (Fig. 1). First, a population with strong kin structure, which may often be associated with reduced effective genetic diversity, may competitively reduce the abundance of heterospecific competitors (Yamawo & Mukai, 2020). Secondly, the dominance of a kin–conspecific community may facilitate the re‐domination of the minor (i.e. low‐frequency) genotypes within the population or migration and establishment of non‐kin genotypes from other populations, as kin‐based populations can be more vulnerable to intraspecific competition. Third, the establishment of non‐kin genotypes increases both within‐population genetic variation and the frequency of interactions among unrelated conspecifics. This shift may allow heterospecific competitors to dominate, because non‐kin pair *P. asiatica* have a disadvantage in interspecific competition (Yamawo & Mukai, 2020). Finally, if a subgroup of siblings forms within a population dominated by heterospecific competitors, strong interspecific competition may reshape population genetic structure through differential fitness among genotypes, potentially resulting in a more kin‐structured population and reduced genetic diversity, as observed at the outset.

**Fig. 1:**
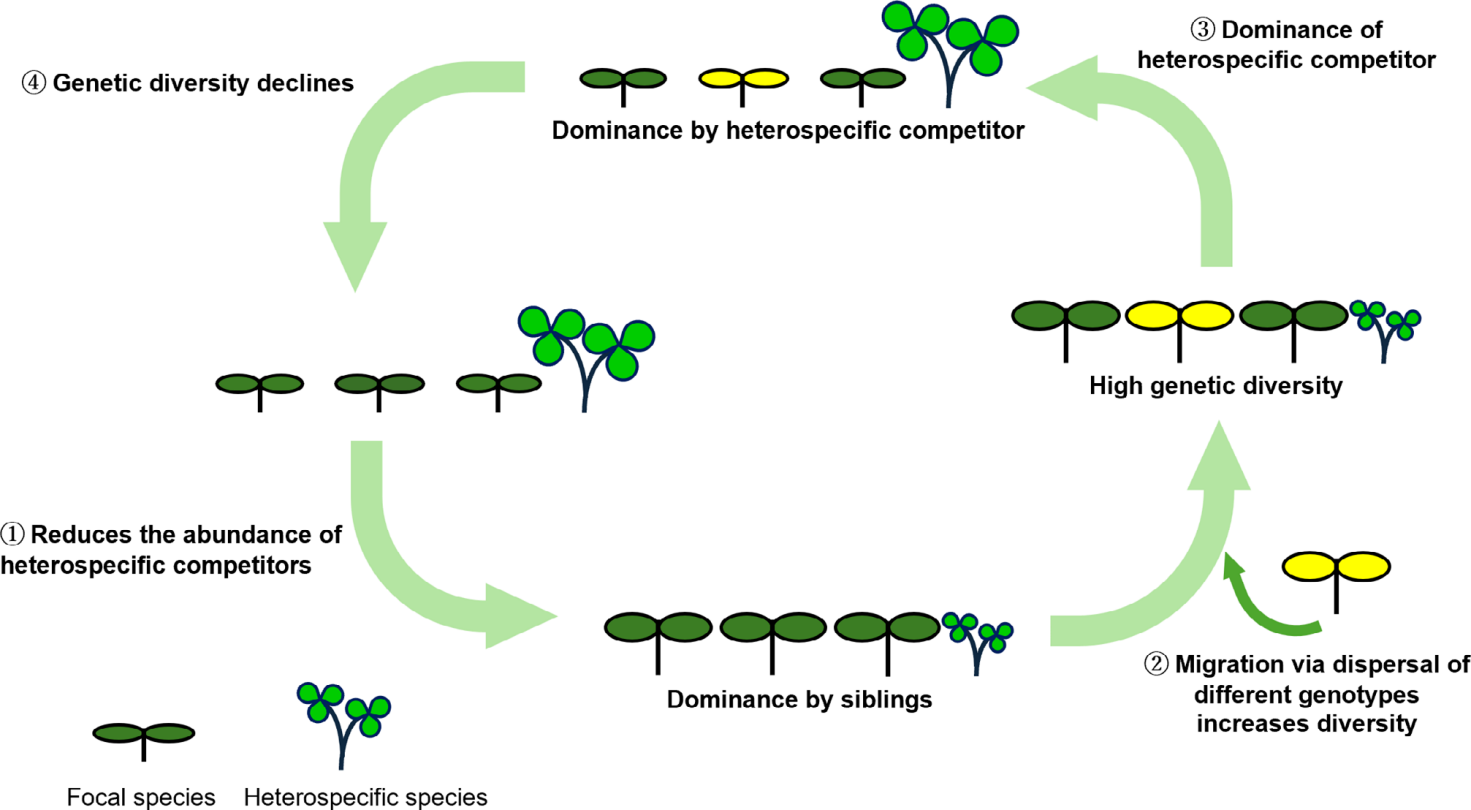
Hypothetical effects of kin discrimination on population‐ and community‐level interactions in *Plantago asiatica*. ①A population dominated by siblings, and thus characterized by strong kin structure and potentially reduced within‐population genetic diversity, reduces the abundance of heterospecific competitors (Yamawo & Mukai, 2020). ② The dominance of a kin‐conspecific community may facilitate the re‐domination of the minor genotype within the population or migration and establishment of non‐kin genotypes from other populations, as kin‐based populations can be more vulnerable to intraspecific competition. ③ The incorporation of non‐kin genotypes increases within‐population genetic variation and the frequency of interactions among unrelated conspecifics, which may reduce the competitive advantage of *P. asiatica* over heterospecifics and allow heterospecific competitors to increase in abundance (Yamawo & Mukai, 2020). ④ Finally, if a subgroup of siblings forms within a population amid the dominance of a heterospecific competitor, strong interspecific competition may favour the particular genotypes or kin groups,  thereby reshaping the genetic structure of the population and potentially resulting in a more kin‐structured population with reduced effective genetic diversity, as seen at the outset. Feedback between the effects of reduced competition among related conspecifics are expected to affect the strength of intra‐ and interspecific competition and both local‐ and community‐level diversities. The relative sizes of plants indicate hypothesized relative plant abundance.

Although such a complete cycle is likely to occur only under specific ecological conditions, for example, in plant species with limited seed dispersal and spatially structured populations, it conceptually illustrates how kin structure, migration, and interspecific competition could interact to shape genetic diversity and community composition. In reality, these processes may also occur locally within populations, and the spatial scale at which they operate could lead to diverse impacts on overall community structure. In addition, such community dynamics are also likely to be mediated by belowground mutualisms, such as mycorrhizal associations, because kin discrimination has been shown to influence the abundance and composition of symbiotic microbial partners (File *et al*., 2012; Asay *et al*., 2020), potentially amplifying or buffering competitive outcomes. Future research, including mesocosm and field studies, is needed to understand how these responses modify long‐term interspecific competition outcomes and influence population and community dynamics. For example, future research should more specifically employ the experimental approaches outlined in Fig. 2 to elucidate the conditions under which identity discrimination affects competitive interactions and community structure. In addition to assessing the generality of this phenomenon, such studies must carefully examine the mechanistic pathways by which it arises, including niche expansion or contraction, changes in competitive ability, and other demographic or ecological processes, in order to evaluate the potential functions of kin discrimination in shaping plant community structure.

**Fig. 2:**
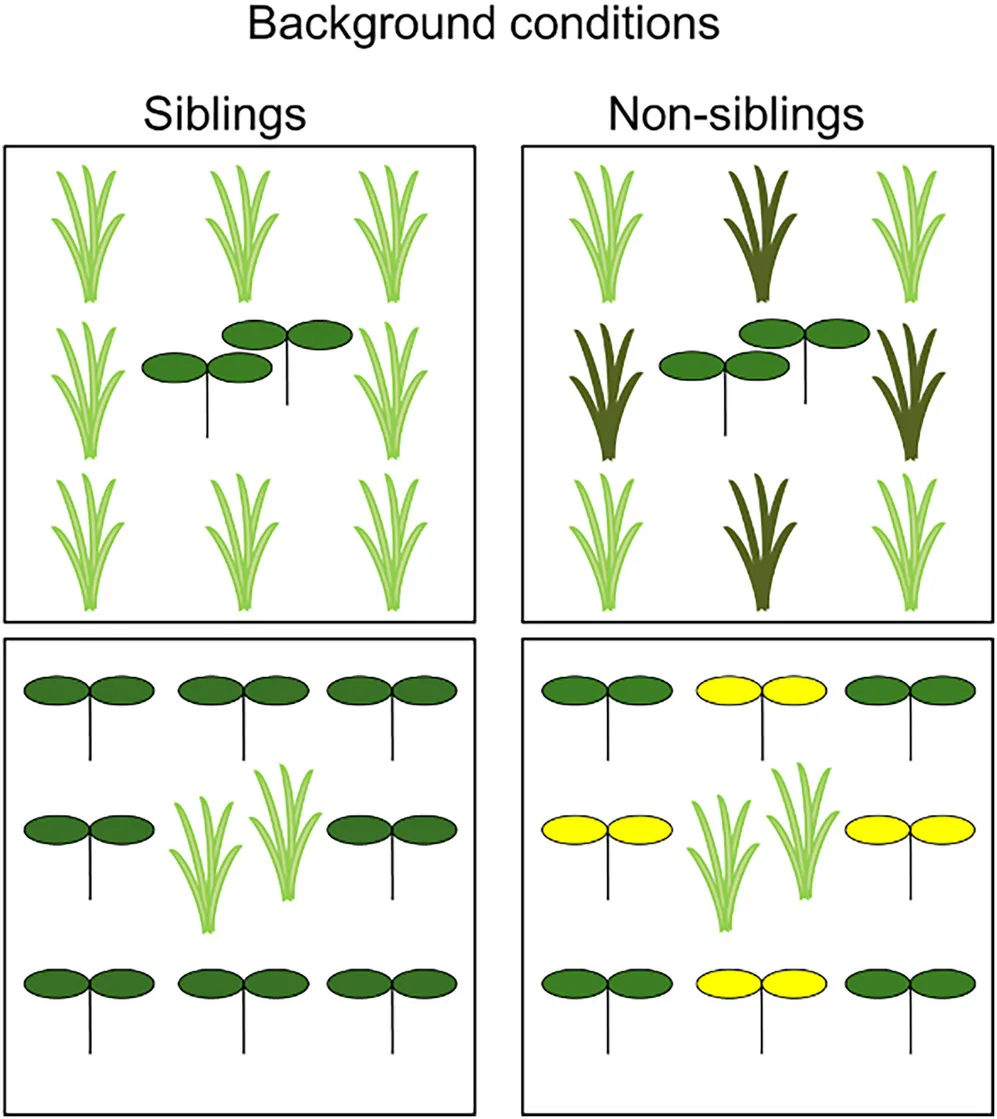
To evaluate the role of kin discrimination in plant communities, focal plants can be introduced as related or unrelated pairs. Comparing invasion dynamics between these treatments allows assessment of how kin discrimination contributes to niche differentiation and fitness equalization. Manipulating the planting density of background heterospecifics to estimate competition coefficients further enables a quantitative evaluation of whether kin discrimination affects the outcome of interspecific competition. In addition, varying the genetic relatedness of background individuals or incorporating spatial structure into the design, such as by changing the distance between focal and background plants, can reveal how social and spatial contexts modulate the effects of kin discrimination on competitive interactions. Different shapes indicate different species whereas different colours represent genetic diversity within the same species.

As mentioned above, kin discrimination in plants demonstrates potentially important effects of genetic identity and structure within populations and interactions at the community level. Because intra‐ and interspecific interactions are common forces in plant communities (Novoplansky, 2009; Pierik, Mommer & Voesenek, 2013; Adler *et al*., 2018), neighbour‐genotype–dependent interactions during interspecific competition are likely to occur in many other plant species where siblings and kin grow in close proximity (Yamawo & Mukai, 2020). Future studies combining population genetics and behavioural ecology are needed to unravel interspecific competition and community dynamics in plants (Yamamichi *et al*., 2020).

### (2) Effects on the trophic and mutualistic interactions between plants and arthropods

It has long been recognized that plant interactions influence trophic interactions, including those involving not only arthropods but also mammalian herbivores (Agrawal *et al*., 2006; Ohsaki *et al*., 2022a,b; Gols & Harvey, 2023), and excellent reviews are available on this topic (Agrawal *et al*., 2006; Gols & Harvey, 2023). Kin discrimination in roots affects plant–animal interactions by directly modifying plant competition, shaping plant defence and the impact of herbivory on plant fitness. Variations in competition intensity due to kin discrimination can influence the expression of leaf defence traits against herbivores through resource allocation. The compensatory continuum hypothesis contends that plants allocate more resources to defensive traits under low competition than under high competition, as the costs of anti‐herbivore defences are greater when competition is intense (Karban & Myers, 1989; Maschinski & Whitham, 1989; Siemens *et al*., 2003; Cipollini, 2007, 2010). In contrast, the defence stress benefit hypothesis suggests that plants may invest more in defences under high competition, where these traits can provide additional benefits, such as allelopathic effects or associational defences, thereby offsetting the costs of these defences and enhancing their expression (Inderjit & Del Moral, 1997; Siemens *et al*., 2003; Cipollini, 2007; Lankau & Strauss, 2007; Boege, 2010). If plants allocate greater resources to defence when growing with siblings, this would support the compensatory continuum hypothesis. Alternatively, if plants allocate greater resources to defence when growing with non‐siblings, this would support the defence stress benefit hypothesis. The mechanisms mentioned above are not mutually exclusive and can even occur simultaneously. Researchers can test these hypotheses by estimating the development of defence traits in growth experiments using solitary, sibling, and non‐sibling conditions. These responses are likely underpinned by mechanisms related to the well‐studied trade‐off between defence and shade avoidance (Moreno *et al*., 2009; Ballaré, 2014; Ballaré & Pierik, 2017). By extending these conceptual frameworks, we may gain a better understanding of how kin discrimination shapes plant defence traits.

In a study of the effects of damage to the leaves of *Urena lobata* var. *tomentosa* plants, which have extrafloral nectaries, in kin and non‐kin competition and with ant occupation and removal, more ants visited plants growing with non‐siblings than plants growing alone (Yamawo, 2021). This was because plants growing with non‐siblings had a higher shoot allocation and also secreted more extrafloral nectar than plants growing alone or with siblings. These results suggest that light competition is an important selective force among neighbouring plants and that kin discrimination may function to reduce competitive interactions for light among siblings. Plants without ants experienced greater caterpillar damage to their leaves, regardless of whether they grew with sibling or non‐sibling conspecific neighbours. This pattern highlights the protective role of ants against herbivory. Ant exclusion increased leaf damage more in plants growing with non‐siblings than with siblings (Yamawo, 2021), while no such effect was observed when plants were grown alone. These results suggest that the identity of kinship neighbouring genotypes influences plant reliance on ant–plant mutualism, likely because plants competing with non‐siblings invest less in herbivore defence. Similar patterns were observed in a study on *Mallotus japonicus* (Yamawo, 2015).

Physical and chemical defences are generally more costly than ant‐mediated ones (e.g. Redman, Cipollini & Schultz, 2001; Strauss *et al*., 2002; Vickery & Vickery, 1981; Katayama & Suzuki, 2011; O'Dowd, 1979; Yamawo *et al*., 2015). For example, secondary metabolites can make up 20–30% of a leaf's dry weight, while extrafloral nectar requires only about 1% of leaf energy. Some pioneer plants reduce chemical defences when protected by ants, potentially promoting faster growth (Dyer *et al*., 2001; Yamawo *et al*., 2015). This shift to lower‐cost, ant‐based defence may benefit plants competing with non‐kin by freeing up resources for growth or root development, suggesting that neighbour identity influences investment in mutualistic defence. The existence of other differences among defence‐related leaf traits, such as leaf mass per area and polyphenols in response to neighbouring plants, a large leaf mass per area and more polyphenols in the non‐sibling condition (Takigahira & Yamawo, 2019; Lepik *et al*., 2012), suggests that the influence of kin discrimination is not limited to defensive mutualisms with ants.

The response to the genetic relatedness of neighbours also affects susceptibility to pathogens. In many cases in both rice and wheat, susceptibility or immunity to pathogens is modified by the presence of intraspecific neighbours (Pélissier *et al*., 2021, 2023). Thus, kin discrimination may affect trophic interactions with herbivores and predators of various plant taxa. However, empirical evidence remains limited to a few species, such as *Urena lobata* var. *tomentosa*, as mentioned above (Yamawo, 2021). Further research is needed to understand the broader patterns.

This review suggests that it is reasonable to expect a decrease in genetic diversity within a population due to kin discrimination (Herben & Novoplansky, 2008; Yamawo *et al*., 2017; Fig. 1). On the other hand, herbivory often increases genetic diversity through associational defence effects among different genotypes or spatially varying selection (e.g. Parker, Salminen & Agrawal, 2010; Kotowska, Cahill & Keddie, 2010; Gloss *et al*., 2013; Sato, 2018). When considered alongside the idea that low genetic diversity confers a competitive advantage in interspecific interactions (Yamawo & Mukai, 2020), interspecific competition and herbivory pressure may jointly shape the genetic structure of populations in non‐trivial ways (the competition–herbivory balance hypothesis).

## V. FUTURE DIRECTIONS

Systematic ecological field experiments are needed across different organizational levels, where population‐ and community‐level interactions are explored in multiple taxa in which genetic relatedness is manipulated or accounted for. For example, testing the competition–herbivory balance hypothesis might require an integrated approach combining mesocosm experiments and genetic analyses. Monitoring the genetic relatedness and diversity of plants in natural populations alongside the abundance and biomass of the species will enable estimation of the interactions between genetic diversity of focal plant species and the surrounding plant community. Mesocosm experiments that manipulate the genetic structure of focal plant populations and expose them to interactions with other species communities will help in studying the effects of kin discrimination and prompt novel hypotheses for the implications of these interactions in natural populations and communities. By monitoring natural populations and communities, it may be possible to assess the long‐term impacts of the factors on population and community stability and on species coexistence and diversity. It will be crucial to consider the spatial structure of target plant individuals in these surveys and experiments, because plastic responses based on kin discrimination often require direct interactions and may cause interesting spatial patterns (Herben & Novoplansky, 2008). Most previous studies of the ecological functions of plant genetic diversity have randomized or blocked spatial structure and excluded belowground interactions between plants. However, responses driven by kin discrimination may challenge and alter the conclusions of these studies. In natural ecosystems, plant distribution is often heterogeneous, leading to various identity‐based interactions. Moreover, aggregation is known to affect the outcomes of interspecific competition (Monzeglio & Stoll, 2008; Shinohara & Ohsaki 2025; Gibbs *et al*., 2026). While addressing all such interactions is challenging, kin discrimination serves as a significant framework for understanding the dynamics of plant populations, communities, and trophic interactions.

## VI. CONCLUSIONS

(1) This review highlights the cascading effects of plant kin discrimination on populations and communities. Evidence accumulated so far indicates that such discrimination can influence ecological and evolutionary processes across multiple levels of biological organization.
(2) Current evidence for kin discrimination is disproportionately derived from species with particular life history traits such as short‐lived herbaceous plants. To clarify its generality and ecological significance, broader testing across diverse phylogenetic lineages and ecological contexts is necessary.
(3) Although discrimination among self, kin, and non‐kin likely has profound impacts on population structure, community structure, and trophic interactions, many of these consequences remain insufficiently explored. Experimental and theoretical studies integrating demographic, behavioural, and evolutionary perspectives will be essential for uncovering these mechanisms.
(4) Future research focusing on dominant and long‐lived species, such as trees, can provide key insights into how identity discrimination operates in natural ecosystems. In particular, such research could reveal how kin discrimination contributes to coexistence, cooperation, and competition in complex plant communities.
(5) Ultimately, understanding the diversity, evolution, and ecological functions of plant identity discrimination, including kin discrimination, has the potential to substantially advance our understanding of plant adaptations and their cascading effects across populations, communities, and ecosystems levels.

## Data Availability

Data sharing not applicable to this article as no datasets were generated or analysed during the current study.
